# At the Right Place, at the Right Time. Reply to Vetrugno et al. Comparison between FlowTrac and Pulmonary Arterial Catheter in Off-Pump Cardiac Surgery Patients: “Why Did We Miss Our Appointment?”. Comment on “Oh et al. Comparison between Fourth-Generation FloTrac/Vigileo System and Continuous Thermodilution Technique for Cardiac Output Estimation after Time Adjustment during Off-Pump Coronary Artery Bypass Graft Surgery: A Retrospective Cohort Study. *J. Clin. Med.* 2022, *11*, 6093”

**DOI:** 10.3390/jcm12185772

**Published:** 2023-09-05

**Authors:** Chahyun Oh, Boohwi Hong

**Affiliations:** 1 Department of Anesthesiology and Pain Medicine, Chungnam National University Hospital, Daejeon 35015, Republic of Korea; 2 Department of Anesthesiology and Pain Medicine, College of Medicine, Chungnam National University, Daejeon 34134, Republic of Korea; 3 Big Data Center, Biomedical Research Institute, Chungnam National University Hospital, Daejeon 35015, Republic of Korea

We thank Vetrugno et al. for their interest and comments [1] on our study. I was very interested in reading the article published by your research group [2]. The study was conducted carefully throughout the entire intraoperative period as well as during the postoperative period. The results of the study showed that there was a bias of −0.50 ± 1.72 L/min and an error rate of 37.0% for the second generation Vigileo/FloTrac system when compared to intermittent thermodilution cardiac output (ICO) as the reference. I greatly admire the efforts of your group to conduct such a sophisticated study during dynamic cardiac surgery. With all due respect, I would be happy to provide a point-by-point reply to your comments.

First and foremost, I would like to emphasize the main concept of our study [3]. Previous studies, including your own, which compared continuous thermodilution cardiac output (CO_cont_) with other methods, did not take into consideration the time delay of CO_cont_, which is known to have a delay of approximately 4 to 12 min [4]. It is necessary to have the “right time” to match CO_cont_ with the test method. To derive optimal time adjustments, we utilized cross-correlation analysis between the two methods, although it was not conducted in an exhaustive manner. In addition, we aimed to validate the performance of CO-FloTrac using continuous data, rather than intermittent or experimental settings (i.e., at pre-specified time points or before and after an experimental maneuver, or only after achieving steady state). Such experimental settings may not fully reveal the realistic performance of the test method.

Second, we agree with your point that specific surgical phases and accompanying manipulations could affect the accuracy of the measurements. To address this concern, we reviewed the cases included in the study and divided the dataset into two subsets, namely, the “harvesting phase” and the “grafting phase”, based on the time point of systemic heparinization. Although this differentiation may not be completely distinctive, the harvesting phase can be considered a relatively stable period without significant cardiac manipulation. We repeated the main analysis, including the Bland–Altman and four-quadrant plot analyses, using each subset with individual time adjustments. The results are presented as follows:

1. Harvesting phase:

The bias of CO-FloTrac was −0.75 (95% CI, −1.14 to −0.37) L/min and the PE was 57.6% (95% CI, 44.2 to 76.8%). Depending on the time scale and the size of the exclusion zone, concordance rates ranged from 64.1 to 76.5%.

2. Grafting phase:

The bias of CO-FloTrac was −1.05 (95% CI, −1.49 to −0.60) L/min and the PE was 70.0% (95% CI, 55.4 to 91.0%). Depending on the timescale and the size of the exclusion zone, concordance rates ranged from 61.6 to 74.9%.

As you pointed out, we did find that the performance of CO-FloTrac was better in the harvesting phase compared with the grafting phase. It appears that our previous result in the study is somewhere in the middle. 

Third, is it possible to consider CO_cont_ as a standard? While many in the field consider ICO as the gold standard for CO measurement, there are clinicians who disagree [5], including myself. This is because ICO measurement is prone to human error, such as injection rate and experience, and is cumbersome to perform during dynamic intraoperative periods. This is why I believe that CO_cont_ is a more practical standard. A recent meta-analysis has also shown that CO_cont_ meets the criteria for interchangeability with ICO [6].

Fourth, regarding the 30% error cutoff for interchangeability, it is worth considering the precision of the reference method. Since this study was retrospective, we could not directly measure the precision of the reference method (CO_cont_) and had to rely on a value from a previous study. Specifically, we used a 30% pooled percentage error from a meta-analysis comparing ICO and CO_cont_ [6]. Given that the precision of ICO is generally considered to be 20%, we assumed the precision of CO_cont_ to be 20% as well, resulting in a combined precision of 28.3% ($\sqrt{20^{2}+20^{2}}$). If both the reference and test methods have a precision of 30%, then the cutoff could be extended to approximately 45% ($\sqrt{30^{2}+30^{2}}$ = 42.4). As noted by Peyton et al. [7], the criterion of 30% may not be suitable in clinical practice, particularly in cardiac surgery, as the precision of the reference method (thermodilution) could be compromised intraoperatively (not in the “right place”). While this may be a more realistic expectation, the results of our study did not meet the extended cutoff either. In general, minimally-invasive devices for CO monitoring are not considered to be precise based on either criterion [8].

Lastly, regarding the results of the patients with arterio-venous fistula (n = 2) and EF < 40% (n = 2), I found several articles on these issues. Although direct evidence regarding the performance of CO-FloTrac in patients with arterio-venous fistula is scarce, it may be compromised due to altered vascular compliance and tone [9,10]. Maeda et al. assessed the performance of CO-FloTrac compared with 3D echocardiography in patients with EF ≤ 35% [11]. The overall bias was 0.60 ± 0.63 L/min with a high percentage error of 58.2%. In one of the two cases with EF < 40% in our study, CO-FloTrac showed sound performance, while in the other, it did not. Since this issue was not the primary concern in our study, no valid conclusion can be drawn. However, we still believe that advanced hemodynamic monitoring devices should be tested with patients with various comorbidities.
